# Medical doctors' job specification analysis: A qualitative inquiry

**DOI:** 10.3205/zma001120

**Published:** 2017-10-16

**Authors:** Anike Hertel-Waszak, Britta Brouwer, Eva Schönefeld, Helmut Ahrens, Guido Hertel, Bernhard Marschall

**Affiliations:** 1 Universität Münster, Medizinische Fakultät, Institut für Ausbildung und Studienangelegenheiten (IfAS), Münster, Germany; 2 Universität Münster, Institut für Psychologie, Lehrstuhl für Organisations- und Wirtschaftspsychologie, Münster, Germany

**Keywords:** qualitative workplace requirements analysis, medical competencies, medical student selection, competence development, medical education, potential appraisals

## Abstract

**Purpose:** A qualitative inquiry was conducted to investigate the qualification requirements of medical doctors in different professional fields and from different perspectives. The inquiry was part of an empirical workplace analysis.

**Methods: **Seventy-four structured interviews were conducted and analyzed to examine critical incidents and behaviors of medical doctors working in different professional fields (clinical theory, clinical practice, practitioner) and disciplines, and from three different perspectives (medical doctors, non-medical staff, and patients). In addition, the National Competency-based Catalogue of Learning Objectives for Medical Education (Nationaler Kompetenzbasierter Lernzielkatalog Medizin / NKLM) was used.

**Results: **The results revealed eleven relevant competencies, which could be categorized into three superordinate competence clusters: interpersonal, work-related, and self-related. The perspectives of medical doctors and non-medical staff included all eleven competencies. However, the perspective of patients did not include one interpersonal and two self-related competencies. Nearly all of the critical behaviors mentioned are included in the NKLM. However, the NKLM also includes behaviors that were not mentioned in the interviews.

**Conclusions: **The behavior-oriented interviews resulted in a requirement profile that is very similar in structure to other competency models in occupational contexts. Comparisons of the different perspectives predominantly revealed similarities. However, the patient perspective also revealed interesting differences compared to the perspectives of medical doctors and non-medical staff. The behavior-related results of the interviews can be directly used for the development of exercises in selection and personnel development contexts and for potential appraisals specific to different medical disciplines. In future steps, the results of this initial qualitative step are to be replicated and extended using quantitative studies and a representative sample. The main overall objective is the definition of relevant competencies both for the selection and development of medical students and for the design of potential appraisals as part of personnel development programs in different medical disciplines.

## Zusammenfassung

**Zielsetzung: **Mit einer qualitativen Erhebung wurden die Anforderungen an Ärztinnen/Ärzte in unterschiedlichen Arbeitsbereichen und Fachgebieten sowie aus unterschiedlichen Perspektiven erfasst. Diese Erhebung fand im Rahmen eines arbeitsplatzanalytisch-empirischen Ansatzes der Anforderungsanalyse statt.

**Methodik: **Es wurden 74 leitfadengestützte Interviews zu erfolgskritischen Ereignissen und Verhalten bei der Arbeit als Ärztin/Arzt aus verschiedenen Bereichen (klinisch-theoretisch, klinisch-praktisch, niedergelassen) und Fachgebieten und aus drei unterschiedlichen Perspektiven (Ärztinnen/Ärzte, nicht-ärztliches Personal, PatientInnen) geführt und analysiert. Auch der Nationale Kompetenzbasierte Lernzielkatalog Medizin (NKLM) wurde integriert.

**Ergebnisse:** Die Ergebnisse dieser qualitativen Phase ergaben elf relevante Anforderungen, die wiederum in drei übergreifende Bereiche zusammengefasst werden können: interpersonale, arbeitsbezogene und selbstbezogene Kompetenzbereiche. Die ärztliche Perspektive und die Perspektive des nicht-ärztlichen Personals enthielten jeweils alle elf Anforderungen. Bei der PatientInnen-Perspektive fehlten eine interpersonale und zwei selbstbezogene Kompetenzen. Nahezu alle Verhaltensweisen sind auch im NKLM enthalten, umgekehrt enthält der NKLM auch Verhaltensweisen die sich nicht in den Ergebnissen der Interviews wiederfanden.

**Schlussfolgerung: **Die verhaltensbasierten Interviews ergaben ein Anforderungsprofil, das in seiner Struktur sehr gut zu anderen Kompetenzmodellen für berufliche Tätigkeiten passt. Zwischen den unterschiedlichen in den Interviews befragten Berufsgruppen ließen sich überwiegend Gemeinsamkeiten feststellen. Die Perspektive der PatientInnen zeigte jedoch auch interessante Unterschiede zu den Perspektiven der Ärzte/Ärztinnen und des nicht-ärztlichen Personals. Die verhaltensbasierten Aussagen aus den Interviews können unmittelbar für die Gestaltung von Übungen für Auswahl- und Entwicklungskontexte sowie für fachgebietsspezifische Potenzialanalysen genutzt werden. In zukünftigen Schritten sollen die Ergebnisse der qualitativen Phase in Form einer quantitativen Studie mit einer repräsentativen Stichprobe repliziert und erweitert werden. Das leitende Ziel ist die Definition von relevanten Kompetenzen sowohl für die Auswahl und Entwicklung von Studierenden der Medizin als auch für die Gestaltung von Potenzialanalysen im Rahmen der Personalentwicklung in verschiedenen medizinischen Fachgebieten.

## 1. Introduction

Research into the knowledge, abilities, skills, and attitudes needed by physicians to successfully do their work is not a new endeavor. Previously published analyses of physician requirements have been based primarily on experience-oriented qualitative or theoretical approaches. Experience-based qualitative approaches use expert surveys, for instance, in the form of workshops or interviews (see [1], [2], [3]). As starting points, theory-based approaches use existing taxonomies such as those for interpersonal skills [4], [5] and concepts such as emotional intelligence [6], or they base themselves on descriptions of the desired moral attitudes of physicians [7], [8], [9]. However, validation studies of these theoretical approaches (e.g., Libbrecht et al. [6], Lievens and Sackett [5], and Lievens [4]) show that, despite the significant effects of the assumed constructs, a significant percentage of the variance in the examined criteria still needs to be accounted for (e.g., the evaluation of interpersonal skills during university study, later professional work as a physician).

In contrast, this study is based on an empirical workplace analysis (see table 1 (Tab. 1)) that has been identified by many researchers, including Schuler [10], as the method of choice for selection and development. As is the case with experience-based qualitative research, the first step includes conducting qualitative interviews, examining relevant documents during the analysis, and consulting experts. This qualitative phase then transitions into a quantitative phase whereby a standardized questionnaire is drafted based on the qualitative analysis. By doing this, it is possible to create a database of critical requirements for medical doctors that lead to sound empirical results. This systematic empirical approach with its robustness and generalizability, combined with its conceptual breadth, makes it more advantageous than purely experience-based qualitative or theory-based approaches.

Initial approaches to empirical workplace analysis are concerned with competencies for successful completion of medical studies. For example, Harris and Owen [11] had 105 survey participants rate 47 statements about non-cognitive requirements according to how important or unimportant they believed these requirements were for new medical students. Reiter and Eva [12] identified seven characteristics through literature research, and 292 survey participants were asked to rank these seven according to the value they felt the characteristics had for studying medicine. In contrast to these previous studies, we focus on medical instead of academic competencies not only to identify new insights for making admissions selections but also to foster the development of medical students. In addition, potential appraisals can be developed into a means of assessing the compatibility between an individual and the medical field.

## 2. Methods

The qualitative survey was administered in cooperation with the Chair of Organizational and Business Psychology at the University of Münster (OWMs) as part of a master’s degree seminar in psychology. The NKLM [13] was taken into consideration alongside the structured interviews described below, which related to critical incidents in medical practice and which followed the critical incident technique [http://www.nklm.de]; the resulting identification of characteristics and competency clusters was also taken into consideration. The NKLM follows an intuitive experience-based approach and describes in detail the roles assumed by physicians. The drafting of this catalogue involved approximately 200 experts and all medical societies and associations (AWMF). After a five-year developmental phase, the NKLM was adopted at the Medizinischer Fakultätentag (MFT) in 2015. As a consequence, the NKLM represents a very rich source of information and a good comparative standard for the qualitative phase of this project.

In this study a total of 74 structured interviews were conducted by 19 trained psychologists (B.A. minimum) in November and December 2015 (see the guideline in the attachment 1 ). The interviewees were medical doctors, non-medical staff, and patients, all from different areas (clinical theory, clinical practice, practitioners) and specialties (see table 2 (Tab. 2)). By selecting these participants, we strove for a degree of representativeness of the medical profession. Examining different perspectives on critical behaviors in the medical profession from insiders and outsiders was intended to also yield a more complete definition of the requirements and explore potential “blind spots” in individual perspectives.

The results of the interviews were compiled in a seminar workshop at the University of Münster. First, all of the critical behaviors in the interview results were identified. These behaviors were then combined into clusters based on psychological principles related to requirement dimensions and competencies. Finally, these competencies were assigned to broader competency clusters (interpersonal, work-related, self-related). These steps were also reviewed and confirmed by physicians.

## 3. Results

Content analysis of behavior during critical incidents (121 in total) was conducted separately for the different perspective groups (physicians, non-medical staff, patients) and across all groups. Analysis of the critical behaviors revealed a profile with 11 different competencies for all perspectives (see table 3 (Tab. 3)). Based on their particular conceptual proximities, the 11 competencies were classified as interpersonal, work-related, or self-related. This classification corresponds very well with existing psychological competency models in other occupational contexts - for instance, in connection with leadership or teamwork (e.g., [14]) and with competency models from educational research ([e.g., [15]). In the case of the latter, Lehmann and Nieke list social competence, procedural competence, competence in dealing with oneself, and competence in dealing with objects as conditional factors for the competence to make decisions and take actions.

Only minimal differences were seen between the three perspective groups regarding the individual competencies. Physicians and the non-medical staff identified behaviors that referred to all 11 competencies. In contrast, behaviors were missing from the point of view of the patients when reflecting on teamwork and abilities to handle stress and criticism.

As part of a seminar at the University of Münster, a comparison of the interview results and the NKLM was also undertaken, whereby two psychologists (one of whom also holds a degree in medicine) independently categorized the interview-based behaviors according to the NKLM. The different critical behaviors were assigned in part to different NKLM content, suggesting potential redundancies in the NKLM itself. After discussing different options for the classifications, the two psychologists cooperatively proposed a solution. Their findings showed that only a few critical behaviors named in the structured interviews could not be found explicitly in the NKLM. These involve the following behaviors: training new employees, praising team members, independently searching for a mentor, doing work that extends beyond a particular scope of responsibility, taking the diagnoses made by other colleagues into consideration, responding in a friendly manner to inquiries from colleagues, being honest/not embellishing facts, focusing completely on the patient in critical cases, not taking advantage of privately insured patients, and implementing adopted changes. The content of the NKLM, in turn, goes far beyond that of the interviews.

## 4. Discussion

Qualitative analysis based on structured interviews resulted in a total of 11 competency requirements for medical doctors. Both physicians and non-medical staff identified each of these 11 competencies. In contrast, the group of patients did not name behaviors that reflect competency in working in teams, handling stressful situations, or accepting criticism. Interestingly, this points to a blind spot in the patient’s point of view. However, it is also conceivable that patients do not describe behaviors that align with such competencies simply because, in their role as patient, they experience situations where these behaviors can be directly observed much less frequently. This means that, from the patient’s perspective, successful decision making and action is based on what is directly visible. A physician’s behavior could very well be directly attributable to requirements not mentioned by the patient and not apparent to the patient. The physician is, for example, only in a position to consider all relevant information in terms of good “information management” if, due to a willingness to engage in teamwork, the physician has successfully obtained this information in advance from other team members. The patient, in many cases, is only able to say that the physician was well-informed during the rounds and was able to synthesize all the relevant information and make it useful.

Naturally, these exploratory results need to be reproduced and further investigated not just in response to the limited number of interviewees or medical specialties investigated in this study. The interview results here are also limited due to the geographic concentration on the region surrounding the German city of Münster. Accordingly, quantitative studies are planned using standardized questionnaires with a larger number of participants and greater representativeness with regard to medical specialties and location. These studies will draw upon established models such as the NKLM and the Fleishman Job Analysis System (F-JAS) [16]. A further limitation of the qualitative phase here is the focus on critical incidents because it is conceivable that competencies can be reflected in uncritical events - for instance, through their frequency. This will be addressed in the quantitative phase and will be expanded upon, as the development of competencies for both routine and specialized tasks is to be assessed.

Overall, we view the results of the qualitative phase, with regard to the different perspectives, as a reason to initially survey physicians only. This is because, compared to the group of patients, they seem better able to assess the requirements of their position.

Applying empirical workplace analysis as an approach to developing requirements in different areas of the medical profession generally offers an array of advantages. During the initial qualitative phase, it ensures that no major behaviors or requirement dimensions are overlooked. Comparison with existing requirement and competency systems can also integrate prior results in overarching theoretical and practical medical framework models. By taking the subsequent quantitative step, a basis for making substantiated claims is then created. Completion of the intended quantitative phase and the identification of general and specialty-specific medical requirements will yield a broad pool of data from which competencies can be derived. A critical issue here involves whether and to what extent the relative importance of the acquired competencies changes over the course of the physician’s medical career. 

For instance, it is conceivable that during university study or upon embarking on a medical career, different competencies are crucial for management positions or for general practice. Discussions must be held on the extent to which medical competencies can be used in the university admission process, as they are supposed to be acquired first over the course of study. Although this appears to be a contradiction, it is not contradictory if student rankings at the beginning of medical study, according to the degree of competency of the major competencies, remains stable over the course of study and if future professional success can be reliably predicted. It is precisely these questions that are addressed by Lievens and Sackett [5]. The results of their study on interpersonal competencies, which relate to forming and maintaining relationships and handling information, provide evidence that student rankings, according to the development of these competencies, hardly change, even after competency training sessions during university study. In this case, selection and development are so closely aligned that the potential of the student admitted to medical school can be used optimally. It must be noted that not all competencies that have been or will be found demonstrate the same degree of stability over time. Instead, it must be assumed that some competencies are easier to learn than those which are more strongly dependent on a student’s personality. In the case of competencies closely related to personality, to which the ability to handle stressful situations could be one, recognition of and reflection on one’s own character-based competencies and the development of appropriate alternative strategies for making decisions and taking actions will play an ever-increasing role over the course of study compared to working on the competency itself. Awareness of this during the selection process could have a significant impact. Deciding how to classify the individual competencies must be clarified in a further step once the overall competency profile and valid competency profile for each medical specialty have been quantitatively determined.

Along with identifying relevant requirement dimensions, the critical incident technique also offers the possibility of using detailed behavior-related descriptions of critical incidents to design methods for selection and personnel development, such as multiple mini-actions (MMA – for an overview, see [17] and [18]), items in situational judgment questionnaires [19], and potential analysis exercises. Making decisions on the nature and design of selection procedures, development programs, and potential analyses must be based on standards such as objectivity, reliability, validity, acceptance, and cost-efficiency [20]. As with earlier studies (e.g. [21], [4], [3]), the usefulness of the procedures used must be verified.

## 5. Conclusions

Qualitative analysis of medical competencies using the critical incident technique and the behavior analyses based on it identified 11 relevant competencies for physicians, which were classified into three different competency areas: interpersonal, work-related, and self-related. The behaviors identified by both physicians and non-medical staff covered all the 11 competency dimensions. The critical behaviors from the point of view of the patients, however, only referred to eight of these competency dimensions. Missing from these competencies were behaviors connected with handling stress, teamwork, and dealing with criticism. In general, approaching workplace requirements analysis using the empirical method applied in this study enables medicine, as an academic subject, to undertake beneficial and promising measures. The developmental and career paths open during and after university study display a high degree of diversity. In this context it is important to differentiate between competencies that are basic to the field and those that are specific to specialties within the field. Identifying the most basic competencies - the smallest common denominator shared by the different medical fields - can be applied to the **selection** of medical students, assuming the existence of stability over time for those competencies. The identification of different, specialty-specific requirements also aids in the advising of students as they pursue different career paths in medicine. As students prepare for the medical profession, both basic and specific competencies play a role in curricula addressing student **development and advising**. Requirement-based **potential analyses** can help physicians-to-be to pursue paths best suited to them personally. A high level of congruence between person and job will significantly and positively affect the performance and satisfaction of the future physician (e.g., [22]). The validity and usefulness of these selected methods must be proven over time. The approach described here can be used to create a good basis for this.

## Acknowledgements

We wish to thank the participants of the Master’s degree seminar on personnel selection during the 2015/16 winter semester taught by Prof. Dr. Guido Hertel at the Department of Psychology of the University of Münster for their support in implementing the qualitative phase of our joint project, namely Da-eun Ahn, Jan Hendrik Beste, Leonie Frank, Eva Götting, Karin Hammeke, Pia Holzgreve, Astrid Janich, Lena Juds, Jennifer Hildebrand, Laura Krebs, Suzana Milicevic, Sophie Morawietz, Antonia Neumann, Jens Paschmann, Christian Pill, Meike Reuter, Lisa Rößler, Olena Skorozhenina, Luisa Tamm, and Sandra Regehr (tutor).

## Competing interests

The authors declare that they have no competing interests. 

## Supplementary Material

Structured Interview: Critical Incident for Practicing Physicians

## Figures and Tables

**Table 1:**
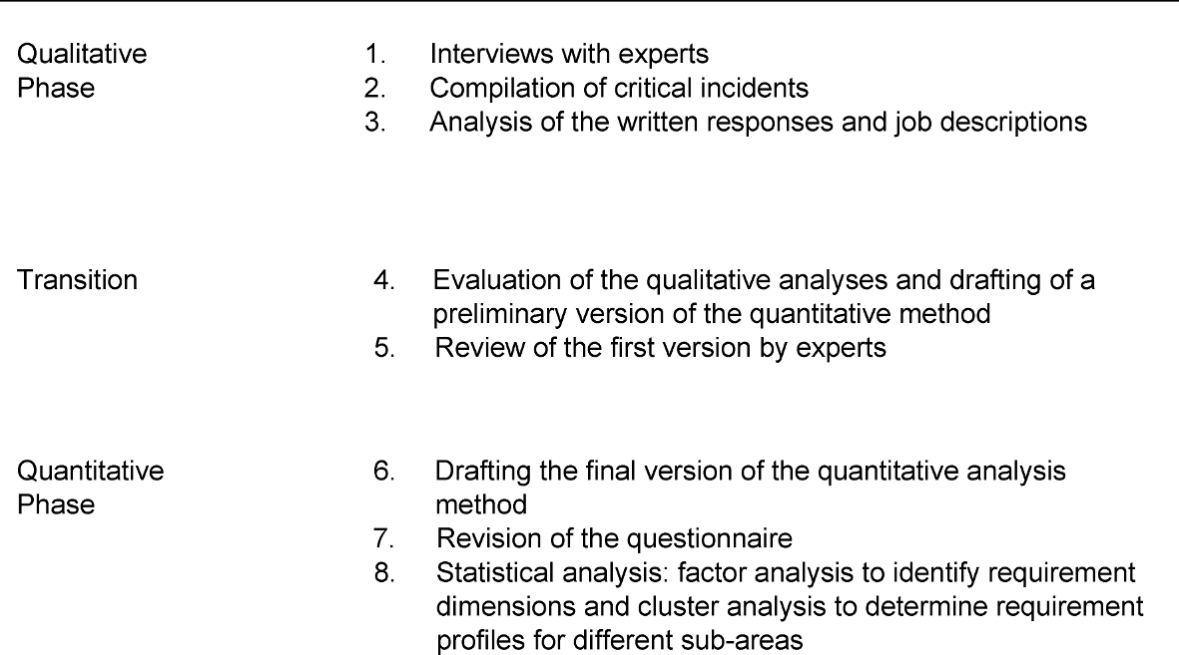
Empirical workplace analysis according to Schuler [10]

**Table 2:**
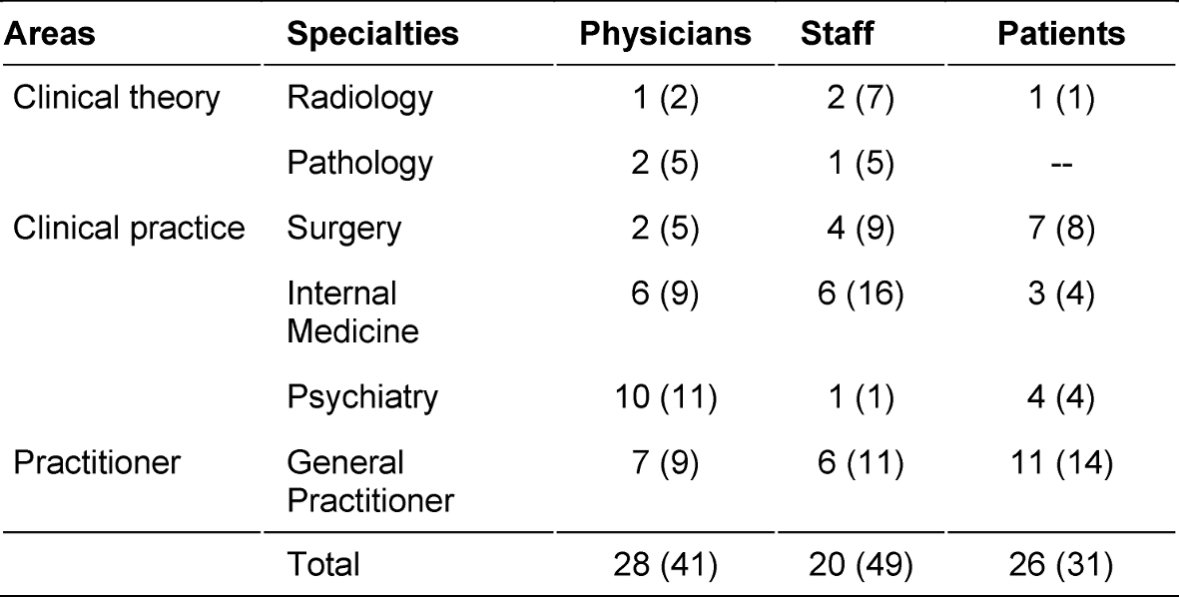
Number of interviews according to fields, specialties, and the three different interview groups (number of critical incidents in parentheses).

**Table 3:**
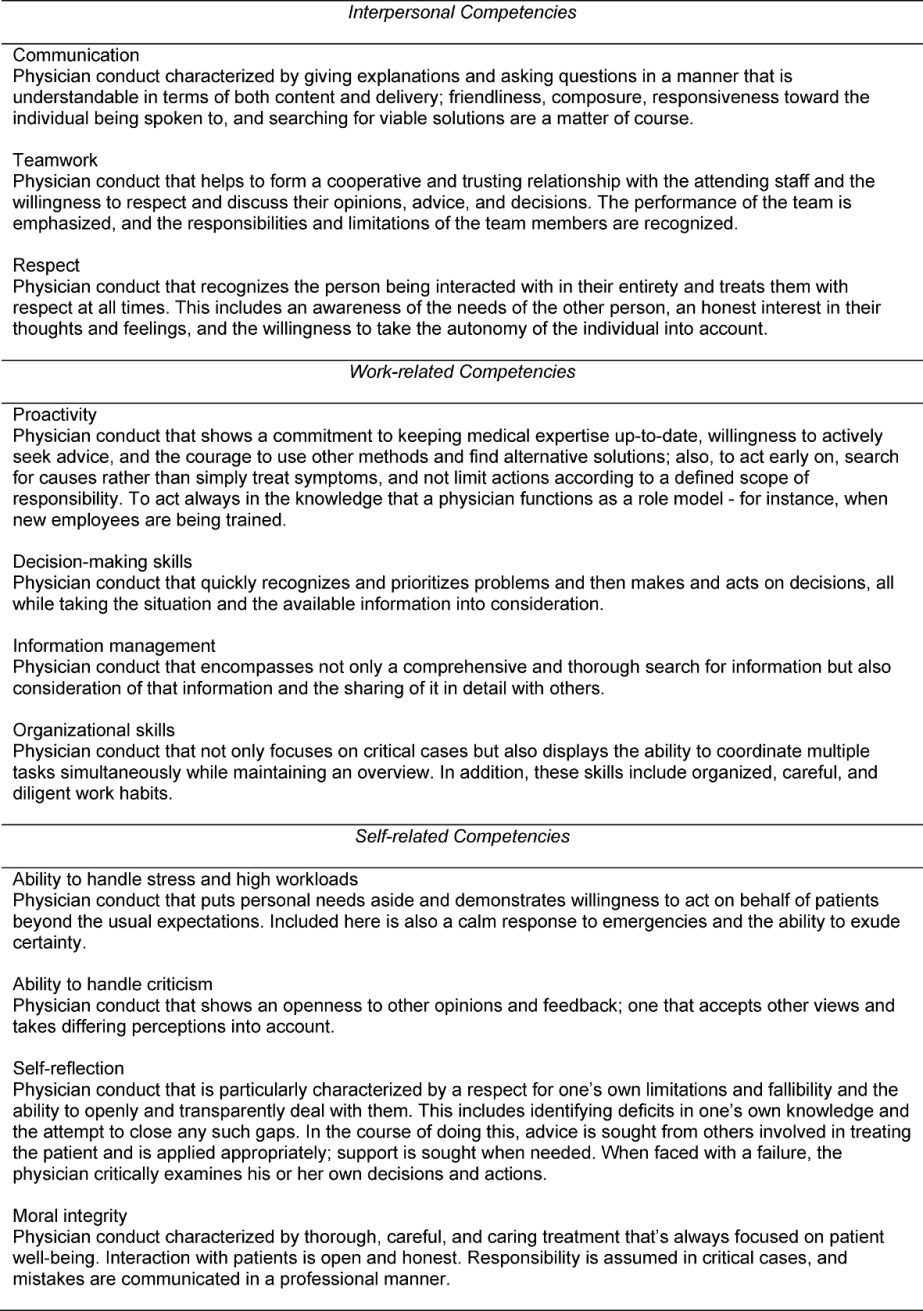
Medical competencies based on critical incidents classified as interpersonal (communication, teamwork, respect), work-related (proactivity, decision-making skills, information management, organizational skills), or self-related (ability to handle stress, ability to handle criticism, self-reflection, moral integrity).
