# Supplementary material for: Medical doctors' job specification analysis: A qualitative inquiry
Source: GMS J Med Educ. 2017 Oct 16;34(4):Doc43. doi: 10.3205/zma001120 (PMC5654118; doi:10.3205/zma001120)
Supplement: Structured Interview: Critical Incident for Practicing Physicians [file JME-34-43-s-001.pdf]

Structured Interview: Critical Incident for Practicing Physicians

Name of interviewer: ____

Interviewee number: _______________ (please number consecutively)

Age: ______ Sex: o Female o Male Group: o Physician o Staff o Patient

Medical field: o Radiology o Pathology o Surgery o Internal Medicine o Psychiatry o General Practitioner

Think of an example that demonstrates particularly effective or ineffective behavior of a medical doctor. Please describe this situation and the behavior as specifically as possible.

• What were the circumstances or background context that led to this behavior?

• Describe the specific behavior of the physician. What was particularly effective or ineffective about this behavior?

• What were the consequences of this behavior?

• What would be normal behavior in such a situation? (How do physicians usually act?)

• What would be particularly *ineffective* behavior in such a situation? (If ineffective behavior has been described, what would be particularly *effective* behavior in such a situation?)

➔ Detailed notes
